# The coordinate systems in the Hess screen test and the Harms tangent screen test for ocular motility analysis of strabismus: their metrics and relationship

**DOI:** 10.1016/j.zemedi.2026.01.006

**Published:** 2026-02-05

**Authors:** Theo Oltrup, Marvin Bende, Thomas Bende, Martin A Leitritz, Karl Ulrich Bartz-Schmidt

**Affiliations:** Division of Experimental Ophthalmic Surgery and Refractive Surgery, Dept. of Ophthalmology, University of Tuebingen, Germany

**Keywords:** Strabismus, Hess screen test, Harms tangent screen test, Coordinate system, Metric tensor, Coordinate transformation

## Abstract

In the diagnosis of strabismus due to paralysis of the eye muscles, the Hess screen test and the Harms tangent screen test are used to measure the misalignment of the eyes in different gaze directions (motility analysis). Both examination methods use different procedures to define the gaze direction and coordinate systems. Coordinate systems with scales in angular degrees are used to determine the direction of gaze and simplify reading the squint angle in the event of a misalignment (horizontal and vertical deviation). In the measurement results, however, their numerical values differ due to the different angle definitions of the direction of gaze in the Hess and Harms tests. In this paper, the angle definitions are clearly described, the coordinate systems are mathematically derived from them, their metrics are formally analysed and compared with the surface theory according to Carl Friedrich Gauss, and their transformation relationship is specified and visualised. The surface theory is used to justify the finding that a measured misalignment of the eye leads to the same motility disorder with the same spatial orientation of the direction of gaze, regardless of the choice of angle. The coordinate systems according to Harms and Hess are therefore mathematically equivalent. However, assessment of the paretic eye muscle(s) and the subsequent therapeutic measures are based on the numerical values of the squint angle measurement. Therefore, knowledge of the test used and its angle definition is important. The procedure for determining the gaze direction determines the choice of coordinate system in the test according to Harms and Hess to avoid systematic measurement deviations.

## 1 Introduction

In the diagnosis of strabismus due to a disturbance in the balance of the extraocular muscles or incorrect motor coordination of the eyes, the angle of deviation (defined as the misalignment of the visual axes of both eyes) under dissociation must be measured in at least 9 gaze directions (motility analysis). This allows a judgement to be made between a paretic strabismus (i.e. a change in the strabismus angle depending on the gaze direction) and a concomitant strabismus (i.e. the same strabismus angle), as well as to identify the eye muscle concerned [1], [2]. Two common examination procedures performed in free space with subjective information from the proband are the Hess screen test [3] and the Harms tangent screen test [4]. Walter Hess [5], [6] first presented his motility scheme in 1908 and described how the paralysed muscle can be identified in a differential diagnosis in the direction of gaze with monocular fixation of the affected eye compared to fixation with the other eye. The direction of gaze is defined by moving the eyes to a predetermined fixation mark while the head is fixed in the direction of the centre of a screen. The test distance to the proband is 0.5 m. In 1941, Heinrich Harms [7] published his motility scheme for a distance of 2.5 m. Here, too, paresis is identified by differential diagnosis. According to Harms, the direction of gaze is achieved by lateral head rotation when the eye is fixed on the centre of its screen. Gernet [8] interprets this method as the only correct one for avoiding systematic errors in squint angle measurement. Both examination methods use coloured glasses and complementary coloured markers for dissociation of the eyes. A coordinate grid with degree scaling is used to determine the gaze direction and simplifies reading the squint angle in the event of a misalignment of the eyes (horizontal and vertical deviation). Harms introduced a coordinate system with straight horizontal and vertical lines, whose scales determine the value of the squint angle according to a tangent function. These are easy to construct and were previously presented by Hirschberg [9] and Sattler [10], for example. According to the Hessian method, these are curvilinear coordinate lines that form rectangles as boundary lines, which follow from the projection of spherical squares [6]. In contrast to Harms, Hess does not go into detail about their construction. While a trigonometric equation of the Hess coordinates is given by Mehringer et al. [11] and Oltrup et al. [12], it is falsifiable if this formula is compared with the metric of a Hess screen. However, if a motility disorder from these two examinations or another examination method is to be compared [13], [14], a correct definition is required, because an analysis of the measurement results is only valid with prior transformation between the coordinate systems. If new examination methods are applied that use other coordinate systems, it is also essential to convert them to the coordinates according to Hess or Harms to which the examiner is familiar.

The aim of this paper is to mathematically represent the coordinate systems according to Hess and Harms. For the Hess system, there appears to be either no literature with a mathematical representation or only literature that is difficult to access. However, this is by no means intended as a value judgement on the non-existence of a publication. We describe the geometry of the coordinate systems using the surface theory of Carl Friedrich Gauss and compare the differences and commonalities. A transformation relation between the coordinate systems is detailed. Finally, the hypothesis of Gernet [8], that only the motility scheme according to Harms results in error-free measurement of the squint angle, is discussed.

## 2 Fundamentals and methods

### 2.1 Definition of direction of gaze

**Fig. 1:**
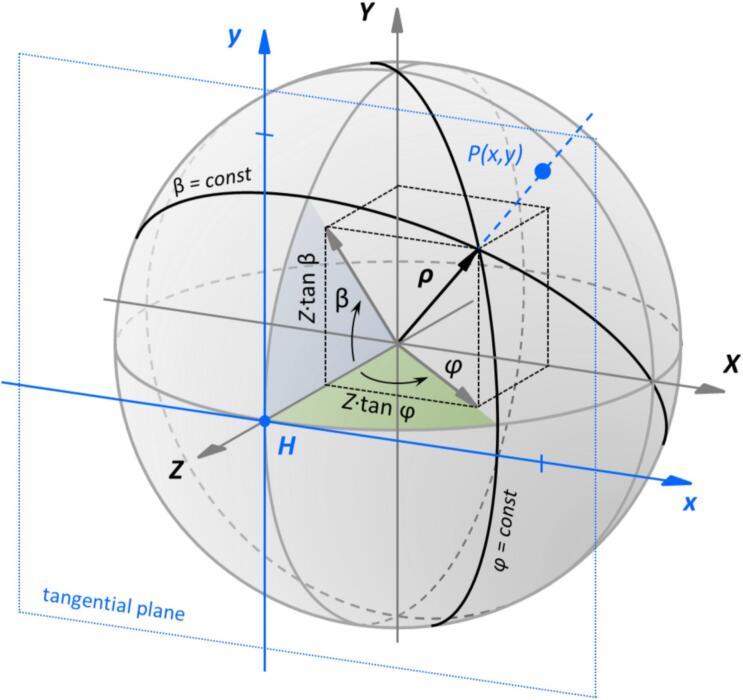
Definition of the gaze direction *ρ* of an eye with the angles (*φ*, *β*) to a hemisphere and its projection *P*(*x*, *y*) into a tangential plane at point *H*. The primary gaze direction is the *Z*-axis.

**Fig. 2:**
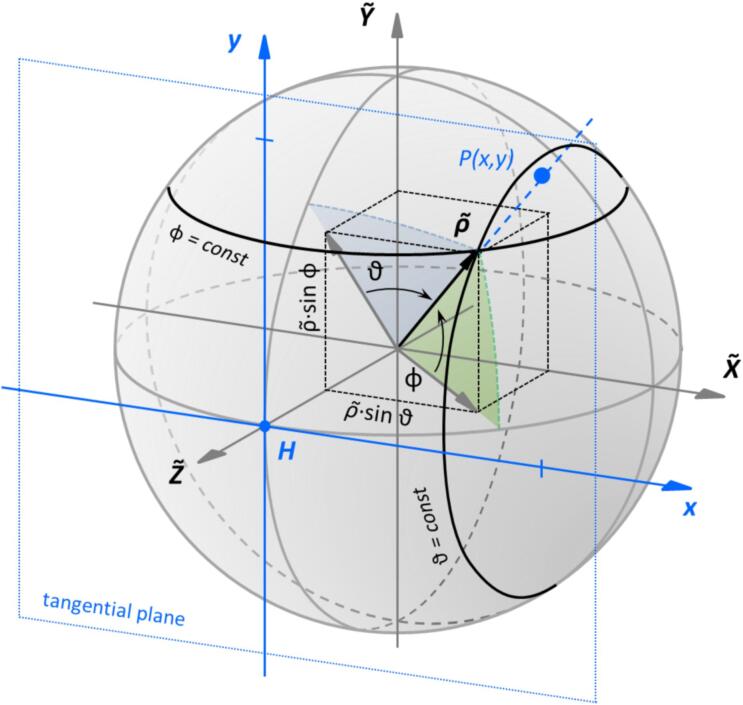
Definition of the gaze direction *ρ̃* of an eye with the angles (*ϑ*, *ϕ*) to a hemisphere and its projection *P*(*x*, *y*) into a tangential plane at point *H*. The primary gaze direction is the Z-axis.

An ophthalmological prism (in its simplest form, a wedge prism) is used for diagnostic and therapeutic purposes in eye misalignment cases [15], [16]. An incident beam of light propagating through a prism to the eye is deflected to the base of the prism (the thickest edge), and its perceived image on the retina is shifted towards the tip of the prism. The deflection of light is indicated by the power of the prism in prism dioptres (1 prism dioptre = 1 cm at a distance of 1 m) and is related to the angle of deviation by the tangent function. For example, if a prism is held in front of the eye with the base pointing nasally, a vertical line in the object space appears to the eye as a horizontal image offset towards the temple (temporal) and the eye turns sideways towards the prism tip at the angle *φ* to align the fovea with the new position. Conversely, if the prism is held with the base in the chin-side direction (inferior), a horizontal line appears to the eye as a vertical image offset, and the eye rotates with the angle *β* in the forehead-side direction (superior). The power of a prism in a tertiary position can be specified as a horizontal and vertical component [17], [18]. A tertiary image offset, and thus the gaze direction of the eye, is then defined by the intersection of two image lines with the parameters *φ* and *β*. If the eye is viewed as a spherical body, then these image lines span a coordinate grid on the retina, whose surface coordinates (*φ*, *β*) are curved lines on the reference surface. If a hemisphere is assumed in object space at a distance *R* from the eye, then this is a further reference surface for the coordinate grid via the nodal point of the dioptric apparatus. A plane image of this grid of degrees (the Harms or Hess screen) is created from the transverse gnomonic azimuthal projection (i.e. the projection is made from the origin of the hemisphere on a tangential plane whose point of contact coincides with the primary viewing direction). The primary line of gaze is defined as a straight-ahead gaze with a normal head and body posture. This is one possibility for describing the gaze direction with horizontal and vertical angles (*φ*, *β*) against the axis of the primary gaze direction (Fig. 1). Alternatively, the orientation can also be defined by the angles (*ϑ*, *ϕ*) between the line of gaze and the sagittal plane or transverse plane of the eye in the primary direction (Fig. 2). This definition is based on the fact that when the eye changes direction horizontally or vertically, the same distance is travelled in one hemisphere with the same change in angle. The observer then sees a grid of degrees as a square pattern in each viewing direction (see Hess [6]).

### 2.2 Metric of a coordinate system

The first fundamental form of the surface theory according to C. F. Gauss is suitable for the classification of a coordinate system [19], [20], [21]. It describes the geometry of a generally curved surface in Euclidean space (*X*, *Y*, *Z*), depending only on its internal metric relationships, which can be determined by measuring the lengths, areas and angles of the surface. Gauss introduced a parametric representation of the Cartesian coordinates *X* = *X*(*u*, *v*), *Y* = *Y*(*u*, *v*) and *Z* = *Z*(*u*, *v*), so that a surface with the position vector ***R***(*u*, *v*) is itself a unique and continuous function of the two parameters *u* and *v*. Exactly one *u*-line (*v* = *v*_0_) and one *v*-line (*u* = *u*_0_) pass through each point *P*(*u*_0_, *v*_0_) of this surface. These parameter lines cover the surface with a regular coordinate grid. The surface theory is applied here to a Cartesian plane (the screens of Harms and Hess) with ***r***(*u*, *v*) = (*x*(*u*, *v*), *y*(*u*, *v*))^T^. The line element in such a surface is in matrix notation, as follows:

(1) $${\mathrm{ds}}^{2}={\mathrm{dx}}^{2}+{\mathrm{dy}}^{2}=\left(\begin{array}{cc} \mathrm{du} & \mathrm{dv} \end{array}\right)\cdot \left(\begin{array}{cc} E & F\\ F & G \end{array}\right)\cdot \left(\begin{array}{c} \mathrm{du}\\ dv \end{array}\right)$$

The line element is given in the Cartesian coordinate system (*x*, *y*) by the Pythagorean theorem. In the parameter system (*u*, *v*), the matrix *M* with the fundamental quantities *E*, *F* and *G* determines the surface’s metric. The matrix is also known as the metric tensor. The value pairs (*du*, *dv*) are small variations along the *u*-lines and *v*-lines, which here stand for the surface coordinates (φ, β) and (ϑ, ϕ). The fundamental quantities are scalar products of the tangential vectors on these lines, which, as base vectors, define a local position-dependent coordinate system. It is $E={\left(\partial r/\partial u\right)}^{2}$, F=(∂r/∂u)·(∂r/∂v) and $G={\left(\partial r/\partial v\right)}^{2}$. If, for example, the quantity *F* = 0, then the local coordinate system is an orthogonal system, and with *E* = *G* = 1, it is also a normalised system. The diagonal elements of the matrix *M* are scale factors with $\left|E\right|=\sqrt{E}$ and $\left|G\right|=\sqrt{G}$. They indicate the length of the base vectors. If two new parameters (*ũ***,** *ṽ*) are introduced, they describe the same surface point *P*(*u*, *v*) with their own fundamental quantities $\tilde{E},\tilde{F\;}\;\mathrm{and}\;\;\tilde{G}$, and thus the same surface, only if the two independent equations *ũ* = *ũ*(*u*, *v*) and *ṽ* = *ṽ*(*u*, *v*) and their inverse functions exist (transformation rule) [20].

## 3 Results

### 3.1 Coordinate systems according to Harms and Hess and their metrics

Consider a Cartesian coordinate system in Euclidean space with the eye localised at its origin (Fig. 1 and Fig. 2). The primary direction of gaze is postulated to be in the direction of the Z-axis. If the eye fixes a surface point *P*(*X*, *Y*, *Z*) on a hemisphere, the position vector ***R*** = (*X*, *Y*, *Z*)^T^ defines its direction of gaze. In Fig. 1, this vector ***R*** with the length *ρ* is parameterised by the surface coordinates (*φ*,* β*). These are the angles between the primary gaze direction and the orthogonal projections of the vector in the transverse and sagittal planes (*X*, *Z* and *Y*, *Z* planes). In Fig. 2, the vector ***R*** with the length *ρ̃* is parameterised by the surface coordinates (*ϑ*,* ϕ*). These are the elevation angles between the vector and the named planes. The components of the vector are obtained by applying trigonometry and the Pythagorean theorem, as follows:

(2) $$\begin{array}{c} X=Z\cdot tan\left(\varphi \right)\\ \;\\ Y=Z\cdot tan\left(\beta \right)\\ \;\\ Z=\sqrt{\rho^{2}-X^{2}-Y^{2}}=\rho /\sqrt{1+\tan^{2}\left(\varphi \right)+\tan^{2}\left(\beta \right)} \end{array}$$

(3) $$\begin{array}{c} \tilde{X}=\tilde{\rho }\cdot sin\left(\vartheta \right)\\ \;\\ \tilde{Y}=\tilde{\rho }\cdot sin\left(\phi \right)\\ \;\\ \tilde{Z}=\sqrt{{\tilde{\rho }}^{2}-{\tilde{X}}^{2}-{\tilde{Y}}^{2}}=\tilde{\rho }\cdot \sqrt{\cos^{2}\left(\vartheta \right)-\sin^{2}\left(\phi \right)} \end{array}$$

The tangential plane at point *H* is parallel to the frontal plane (*X*, *Y* plane) of the eye in the primary position. The projection of the point *P*(*X*, *Y*, *Z*) into the plane *P*(*x*, *y*) is therefore easy to specify for central projection [22]: The transformation equations are *x* = |***R***|· *X* / *Z* and *y* = |***R***| · *Y* / *Z*. The Cartesian coordinates are therefore in the projection plane for a general direction of gaze, as follows:

(4) $$r\left(\varphi ,\beta \right)={\left(\begin{array}{cc} \rho \cdot tan\left(\varphi \right), & \rho \cdot tan\left(\beta \right) \end{array}\right)}^{T}$$

(5) $$r\left(\vartheta ,\phi \right)={\left(\begin{array}{cc} \tilde{\rho }\cdot \frac{sin\left(\vartheta \right)}{\sqrt{\cos^{2}\left(\vartheta \right)-\sin^{2}\left(\phi \right)}}, & \tilde{\rho }\cdot \frac{sin\left(\phi \right)}{\sqrt{\cos^{2}\left(\vartheta \right)-\sin^{2}\left(\phi \right)}} \end{array}\right)}^{T}$$

**Fig. 3:**
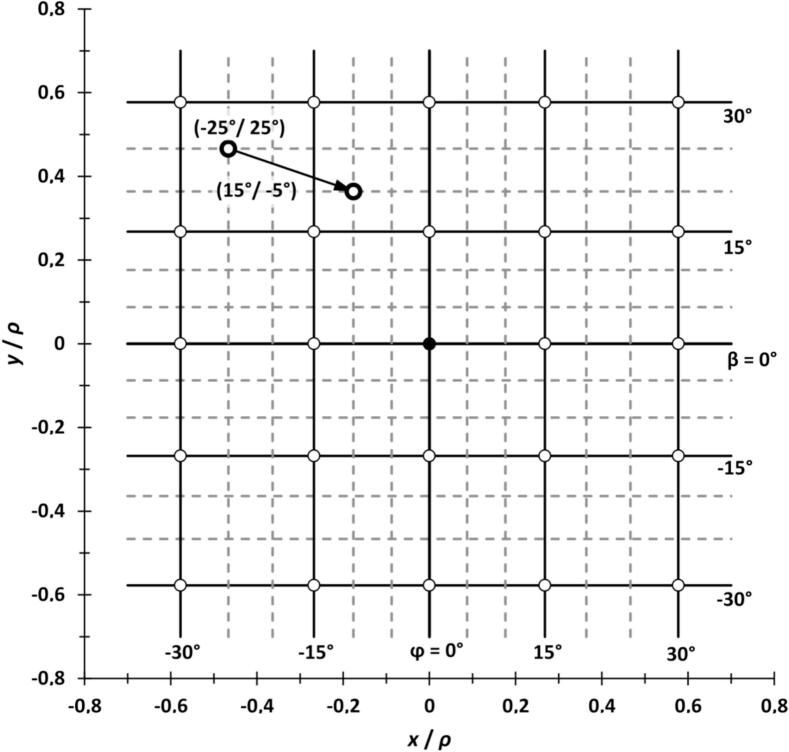
Coordinate system in the Harms tangent screen test. The parameters (*φ*, *β*) on the coordinate lines define the direction of gaze of the eye at a distance *ρ* from the screen in the Harms motility scheme. A deviation (15°/-5°) in the gaze direction (-25°/25°) is illustrated.

**Fig. 4:**
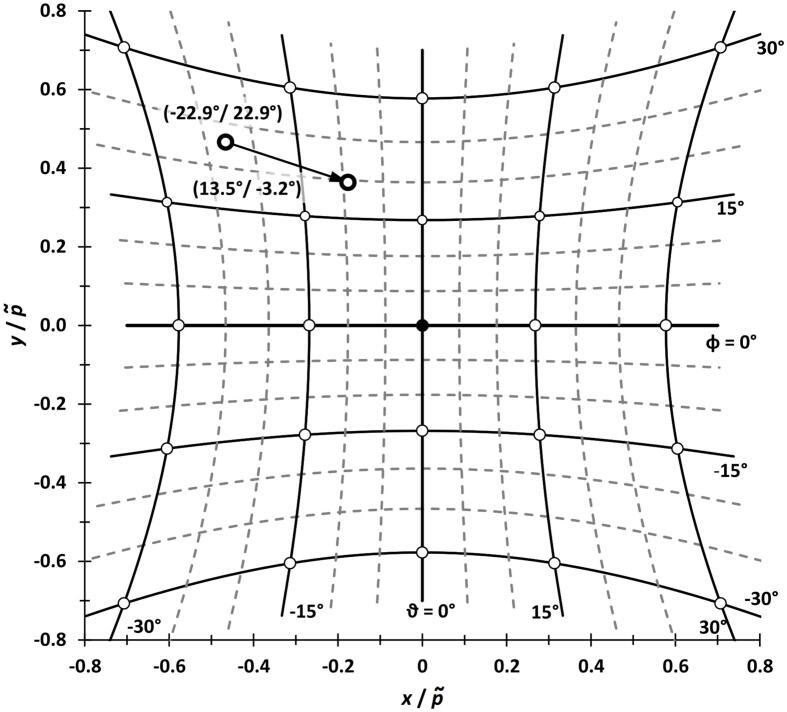
The parameters (*ϑ*, *ϕ*) on the coordinate lines define the direction of gaze of the eye at a distance *ρ̃* from the screen in the Hess motility scheme. A deviation in the direction of gaze is illustrated, which corresponds to the coordinates according to Harms.

A local bijective projection $\left(\varphi ,\beta \right)\mapsto \left(x,y\right)$ based on Eq. (4) is shown in Fig. 3. The parameter lines are identified by the surface coordinates (*φ*, *β*) of the hemisphere with the radius *ρ*. This coordinate system describes the Harms screen [7]. Fig. 4 shows the projection $\left(\vartheta ,\phi \right)\mapsto \left(x,y\right)$ based on Eq (5). Its parameter lines are identified by the coordinates (*ϑ*, *ϕ*) of the hemisphere with the radius $\tilde{\rho }$. This coordinate system describes the Hess screen [5], [6].

The metric tensor *M* of the Harms coordinate system is easy to calculate using the partial derivatives of Eq. (4) and their scalar products, as follows:

(6) $$M=\left(\begin{array}{cc} E & F\\ F & G \end{array}\right)=\rho^{2}\left(\begin{array}{cc} \frac{1}{\cos^{4}\varphi } & 0\\ 0 & \frac{1}{\cos^{4}\beta } \end{array}\right)$$

The metric tensor $\tilde{M}$ of the coordinate system according to Hess requires, in addition to the partial derivatives of Eq. (5) and forming the scalar products, some algebraic transformations. The detailed derivation is presented in the Appendix. The result is as follows:

(7) $$\tilde{M}=\left(\begin{array}{cc} \tilde{E} & \tilde{F}\\ \tilde{F} & \tilde{G} \end{array}\right)={\tilde{\rho }}^{2}\left(\begin{array}{cc} \frac{\cos^{2}\vartheta \cdot \left(\cos^{4}\phi +\sin^{2}\vartheta {\cdot sin}^{2}\phi \right)}{{\left(\cos^{2}\phi -\sin^{2}\vartheta \right)}^{3}} & \frac{sin\vartheta \cdot sin\phi \cdot \left(\cos^{3}\phi \cdot cos\vartheta +\cos^{3}\vartheta \cdot cos\phi \right)}{{\left(\cos^{2}\phi -\sin^{2}\vartheta \right)}^{3}}\\ \frac{sin\vartheta \cdot sin\phi \cdot \left(\cos^{3}\phi \cdot cos\vartheta +\cos^{3}\vartheta \cdot cos\phi \right)}{{\left(\cos^{2}\phi -\sin^{2}\vartheta \right)}^{3}} & \frac{\cos^{2}\phi \cdot \left(\cos^{4}\vartheta +\sin^{2}\vartheta {\cdot sin}^{2}\phi \right)}{{\left(\cos^{2}\phi -\sin^{2}\vartheta \right)}^{3}} \end{array}\right)$$

The fundamental quantity *F* of the metric *M* is zero in the entire definition range of *φ* and *β*. The parameter lines of the Harms coordinate system therefore form a rectilinear orthogonal mesh. The rectilinearity follows from the diagonal elements *E*(*φ*) and *G*(*β*) as functions of only one surface coordinate. The quantity $\tilde{F}$ in the metric $\tilde{M}$, conversely, is a function of *ϑ* and *ϕ*. In the coordinate system according to Hess, the angle *γ*, which includes two parameter lines, follows from the scalar product of the direction vectors with $\cos\left(\gamma \right)=\tilde{F}/\sqrt{\tilde{E}\cdot \tilde{G}}$ and is therefore generally not orthogonal. Only in the major axes with *ϑ* = 0 or *ϕ* = 0 is $\tilde{F}$ equal to zero, and therefore its lines are orthogonal. The parameter lines are hyperbolic curves. This can be illustrated with the definition of conic sections in Fig. 2: For example, if the parameter *ϑ* = *ϑ*_0_, then the direction of the position vector ***R***(*ϕ*) describes a line on the lateral surface area of a mathematical cone with the apex at the origin of the coordinate system, the cone axis in the direction of the $\tilde{X}$-axis and an angle of inclination *ϑ*_0_. The lateral surface area has an intersection curve, the *ϕ*-line, with the projection plane. Its angle of inclination to the sagittal plane is 90°. By definition, the intersection curve is a hyperbola for *ϑ*_0_ < 90° [23]. With the angle *ϕ* = 0 (*ϑ* = 0), the fundamental quantity is $\tilde{E}∼\;1/\cos^{4}\vartheta$ ( $\tilde{G}∼\;1/\cos^{4}\phi$ ) and is equivalent to the quantity *E* (*G*) except for the factors *ρ* and *ρ̃*. This means that the tangential vectors ∂r/∂ϑ∼∂r/∂φ and ∂r/∂ϕ∼∂r/∂β are identical in the major axes of the coordinate systems according to Harms and Hess, and therefore the angles in a secondary position of the eye are identical. They could also have been predicted according to Eqs. (4), (5). Both systems have a tangential scale on the major axes. In Harms’ coordinate system with its rectilinear orthogonal mesh, this applies to the entire definition range. The distance between the scales increases with larger scale values due to the scale factor $\sqrt{E}∼\;1/\cos^{2}\varphi$ (the same applies to *G*, $\tilde{E}$ and $\tilde{G}$). The coordinate systems are therefore not normalised.

### 3.2 Transformation of coordinate systems

The relationships between the angles according to Harms (*φ*, *β*) and Hess (*ϑ*, *ϕ*) are derived from the aspect ratios of right-angled triangles, which are given by the direction vector ***R*** and its components. In Fig. 1 are *X*/ρ_YZ_=(*X*/*Z*)·(*Z*/ρ_YZ_) and *Y*/ρ_XZ_=(*Y*/*Z*)·(*Z*/ρ_XZ_), with the lengths *ρ*(.) of the projections of the vector ***R*** in the named planes. In the right-hand terms of the equations, the quotients are trigonometric functions with the angles *φ* and *β*. The left-hand terms are functions of the angles according to Hess (Fig. 2). Therefore,

(8) $$\begin{array}{c} \vartheta \left(\varphi ,\beta \right)=\arctan(\tan\left(\varphi \right)\cdot \cos\left(\beta \right)),\\ \;\\ \phi \left(\varphi ,\beta \right)=\arctan(\tan\left(\beta \right)\cdot \cos\left(\varphi \right)) \end{array}$$

In Fig. 2 are $\tilde{X}$ / ${\tilde{\rho }}_{\text{XZ}}$~ = ($\tilde{X}$ / $\tilde{\rho }$) · ($\tilde{\rho }$ / ${\tilde{\rho }}_{\text{XZ}}$) and $\tilde{Y}$ / ${\tilde{\rho }}_{\text{YZ}}$ = ($\tilde{Y}$ / $\tilde{\rho }$~) · ($\tilde{\rho }$ / ${\tilde{\rho }}_{\text{YZ}}$). The right-hand terms of the equations are quotients of trigonometric functions with the angles *ϑ* and *ϕ*, and the left-hand terms are functions of the angles according to Harms (Fig. 1). Therefore,

(9) $$\begin{array}{c} \varphi \left(\vartheta ,\phi \right)=arcsin(\sin\left(\vartheta \right)/\cos\left(\phi \right)),\\ \;\\ \beta \left(\vartheta ,\phi \right)=arcsin\left(\sin\left(\phi \right)/\cos\left(\vartheta \right)\right) \end{array}$$

**Fig. 5:**
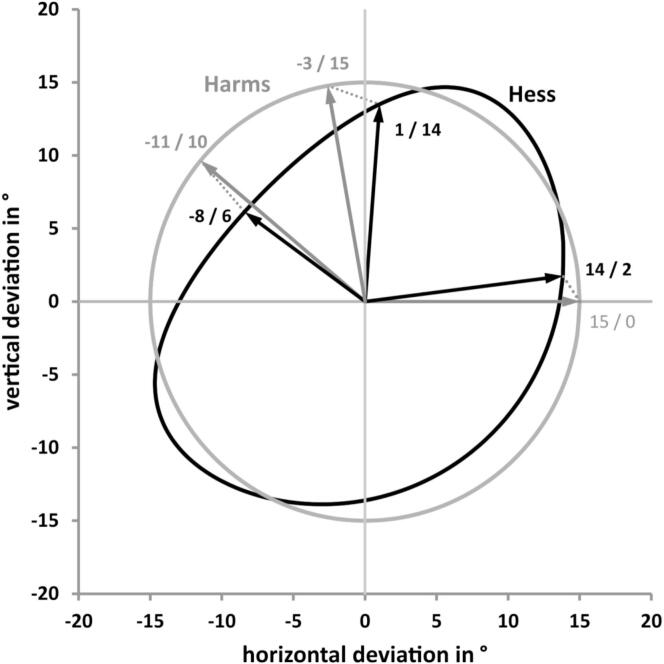
Representation of distortion of a horizontal and vertical deviation between the coordinate systems according to Harms and Hess using the example of a gaze direction (-25°, 25°) and a squint angle of 15° (circle) in the system according to Harms. Three examples of different squint angle directions are illustrated.

By example of a squint angle measurement according to Harms, the transformation into the coordinate system according to Hess is demonstrated. If there is a horizontal and vertical deviation (*Δφ*, *Δβ*) in the given gaze direction (*φ*_0_, *β*_0_), then Eq. (8) is used to calculate this gaze direction with *ϑ*_0_(*φ*_0_, *β*_0_) and *ϕ*_0_(*φ*_0_, *β*_0_) according to Hess. The direction of gaze perceived by the proband at deviation is (*φ*_1_, *β*_1_) = (*φ*_0_ + *Δφ*, *β*_0_ + *Δβ*), which corresponds to *ϑ*_1_(*φ*_1_, *β*_1_) and *ϕ*_1_(*φ*_1_, *β*_1_) in the Hess system. The deviation according to Hess is (*Δϑ*, *Δϕ*) = (*ϑ*_1 _– *ϑ*_0_, *ϕ*_1_ – *ϕ*_0_). Fig. 3 and Fig. 4 show examples of the results of a transformation: In the Harms screen, the deviation (15°, -5°) has been measured in the gaze direction (-25°, 25°), and in the Hess screen, the gaze direction (-22.9°, 22.9°) with the deviation (13.5°, -3.2°) results from the transformation. In Fig. 5, using the transformation rule in the gaze direction (-25°, 25°), a deviation in any direction with the amount 15° (circle) according to Harms is visualised in the Hess coordinates. The projection illustrates the distortion as represented by the Hess coordinates.

## 4 Discussion

The coordinate systems of the tangent screen according to Harms and the screen according to Hess for measuring motility disorders of the eye have been clearly illustrated here and provided in equations. The coordinate systems are plane projections of the surface coordinates of a curved surface (hemisphere) at a given distance from the eye, whose spatial coordinates with two parameters define the surface coordinates and thus the gaze direction. In Harms’s system, the direction of gaze is explained here by the parameters that correspond to the horizontal and vertical angles of a refractive light beam through a prism in front of the eye. In the Hess system, at a hemisphere around the eye as a pendant of the retinal surface, a regular square coordinate mesh is spanned whose projections in the plane correspond to hyperbolic lines. Their parameters are elevation angles to the transverse and sagittal plane of the eye in the primary position (or fixed head position on the centre of the chart in the Hess screen test), which defines the line of gaze. Each coordinate system has its own metric, which is given by a tensor from Gaussian surface theory. The metric tensors were specified and used to analyse the properties of the coordinate system according to Harms and Hess using local lengths and angles on their lines. At their major axes, the systems are orthogonal and equally scaled (tangent scale). The primary and the four secondary directions of gaze, as well as a squint angle value along these directions, are therefore the same in both motility analyses. In the tertiary position, the gaze directions and the horizontal and vertical deviations are different in value and must be transformed when changing the coordinates. The transformation formula was derived. Two other coordinate systems according to Helmholz and Fick, not presented here, are also commonly used in ophthalmology [24]. If the angles according to Harms and Hess are alternated, then the gaze direction is defined with the parameters (*ϑ, β*) according to Helmholtz [25], [26] and with (*φ, ϕ*) according to Fick [27], [28]. The angles based on Helmholtz correspond to the known longitudes and latitudes in the geographical system, while the angles based on Fick correspond to the same system in a transverse position (the polar axis lies in the equatorial plane). Both systems have their own grid of degrees as a plane projection. The Gaussian surface theory ensures that all coordinate systems are equivalent and that a squint angle measurement leads to the same motility disorder. This can also be clearly explained. The measured deviation of an eye on the Harms or Hess screen is a projected directed distance that does not change when the coordinate system is changed (this can be seen in Fig. 3 and Fig. 4 using the example of deviation in the Cartesian coordinates). The directed line is also given by the integral of its line elements according to Eq. (1) in the selected parameter system. However, the line element itself is parameter-invariant; this is where its fundamental significance for surface theory lies. A motility analysis according to Harms and to Hess in the same spatial gaze direction only results in different numerical values for the horizontal and vertical deviation from the choice of angle pairs for the direction definition (see also Fig. 5). Therefore, the choice of coordinate system must be considered in the clinical decision support**,** in order to be able to make a well-founded decision based on it.

However, what is the difference between the two motility schemes if they are equivalent from a mathematical point of view? The choice of coordinate system depends on the method used to define gaze direction. Gernet [8] shows with two illustrated examples on the tangent screen according to Harms that when the gaze is turned sideways and the head is fixed straight ahead to the centre of the screen, the squint angle measurement shows a systematic measurement deviation. For example, if the eye is turned sideways horizontally with (*φ*, *β*) = (30°, 0°), then the measurement distance to the fixation point is *d* = *ρ* / cos(*φ*). A vertical squint angle of *α* = 10° above this line of gaze is then perceived by the proband at the screen position *y* = *d* · tan(*α*) and they see the angle *β* = 11.5° at this position, which is incorrectly specified as 15% in the value of the squint angle *α* [8]. Lateral gaze turns of the eye increase the chosen distance ρ to the screen and thus the position y and its associated squint angle. Gernet concludes that only the motility scheme according to Harms [7] with determination of the lateral gaze direction by turning the head and fixation of the eye to the coordinate origin is error-free. Measurement methods with head fixation and lateral gaze direction of the eye according to Hess [5], [6] are subject to a systematic measurement deviation at the fixation points outside the coordinate origin. However, this conclusion is incorrect. If the measured angle *β* = 11.5° on the tangent screen according to Harms is transformed with *ϕ*(*φ* = 30°, *β* = 11.5°) from Eq. (8), then the result is consequently the assumed squint angle α = 10° according to Hess. The motility scheme according to Hess therefore always leads to the correct squint angle values, regardless of the chosen lateral gaze direction of the eye. The Hessian coordinate grid, as a projection of a regular grid on a hemisphere with a given radius, corrects the unequal measuring distances in the plane through its hyperbolic lines. Therefore, if a measuring method with fixing points outside the coordinate origin is selected, the coordinate system according to Hess is the only correct choice for an error-free squint angle measurement.

The equivalence of the motility schemes is mathematically justified and also applies to different measurement distances (0.5 m for Hess and 2.5 m for Harms) because their transformation equations (Eqs. (8), (9)) are independent of these. However, taking into account the physiology of visual perception, this does not apply without restriction. In binocular vision, the eyes with a parallel position in distant vision lead to opposite movements (convergence) in near vision to bring their directions of gaze to overlap at the fixation point. This can lead to different strabismus tendencies at different distances and must be taken into account when comparing the measurement results of the Harms and Hess tests.

## CRediT authorship contribution statement

**Theo Oltrup:** Writing – review & editing, Writing – original draft, Visualization, Validation, Methodology, Investigation, Formal analysis, Data curation, Conceptualization. **Marvin Bende:** Validation, Conceptualization. **Thomas Bende:** Validation, Supervision, Funding acquisition, Conceptualization. **Martin A Leitritz:** Resources, Conceptualization. **Karl Ulrich Bartz-Schmidt:** Supervision, Project administration, Funding acquisition, Conceptualization.

## Funding

The funds for this work were provided by the Dr. Ernst and Wilma Mueller Foundation. We would like to thank them for their support.

## Declaration of competing interest

The authors declare that they have no known competing financial interests or personal relationships that could have appeared to influence the work reported in this paper.

## Appendix: Derivation of the metric tensor in the Hess coordinate system

With normalised Eq. (5)

$$r\left(\vartheta ,\phi \right)=\left(\begin{array}{c} x\left(\vartheta ,\phi \right)\\ y(\vartheta ,\phi ) \end{array}\right)=\frac{1}{\sqrt{\cos^{2}\left(\vartheta \right)-\sin^{2}\left(\phi \right)}}\left(\begin{array}{c} \sin\left(\vartheta \right)\\ sin(\phi ) \end{array}\right).$$

their direction vectors $a_{\vartheta }:=\frac{\partial r}{\partial \vartheta }=\left(\begin{array}{c} \frac{\partial x}{\partial \vartheta }\\ \frac{\partial y}{\partial \vartheta } \end{array}\right)$ and $a_{\phi }:=\frac{\partial r}{\partial \phi }=\left(\begin{array}{c} \frac{\partial x}{\partial \phi }\\ \frac{\partial y}{\partial \phi } \end{array}\right)$

and the scalar products $\tilde{E}=a_{\vartheta }{\cdot a}_{\vartheta }$ , $\tilde{F}=a_{\vartheta }{\cdot a}_{\phi }$ und $\tilde{G}=a_{\phi }{\cdot a}_{\phi }$ follows (the identity $\cos^{2}\vartheta -\sin^{2}\phi =\cos^{2}\phi -\sin^{2}\vartheta$ applies):

$$a_{\vartheta }=\frac{1}{{\left(\cos^{2}\vartheta -\sin^{2}\phi \right)}^{\frac{3}{2}}}\left(\begin{array}{c} \cos^{2}\phi \cdot \cos\vartheta \\ \sin\phi \cdot \cos\vartheta \cdot \sin\vartheta \end{array}\right)$$

$$a_{\phi }=\frac{1}{{\left(\cos^{2}\vartheta -\sin^{2}\phi \right)}^{\frac{3}{2}}}\left(\begin{array}{c} \sin\vartheta \cdot cos\phi \cdot sin\phi \\ \cos^{2}\vartheta \cdot cos\phi \end{array}\right)$$

$$\begin{array}{cc} \tilde{E}=a_{\vartheta }{\cdot a}_{\vartheta } & =\frac{1}{{\left(\cos^{2}\vartheta -\sin^{2}\phi \right)}^{3}}\left(\cos^{4}\phi \cdot \cos^{2}\vartheta +\sin^{2}\phi \cdot \cos^{2}\vartheta \cdot \sin^{2}\vartheta \right)\\ & =\frac{\cos^{2}\vartheta }{{\left(\cos^{2}\vartheta -\sin^{2}\phi \right)}^{3}}\left(\cos^{4}\phi +\sin^{2}\vartheta \cdot \sin^{2}\phi \right) \end{array}$$

$$\begin{array}{cc} \tilde{F}=a_{\vartheta }{\cdot a}_{\phi } & =\frac{1}{{\left(\cos^{2}\vartheta -\sin^{2}\phi \right)}^{3}}\text{(}\cos^{2}\phi \cdot \cos\vartheta \cdot \sin\vartheta \cdot \cos\phi \cdot \sin\phi \\ & \;+\cos^{2}\vartheta \cdot \cos\phi \cdot \sin\phi \cdot \cos\vartheta \cdot \sin\vartheta )\\ & =\frac{\sin\vartheta \cdot \sin\phi }{{\left(\cos^{2}\vartheta -\sin^{2}\phi \right)}^{3}}\left(\cos^{2}\phi \cdot \cos\vartheta \cdot \cos\phi +\cos^{2}\vartheta \cdot cos\phi \cdot \cos\vartheta \right)\\ & =\frac{\sin\vartheta \cdot \sin\phi }{{\left(\cos^{2}\vartheta -\sin^{2}\phi \right)}^{3}}\left(\cos^{3}\phi \cdot \cos\vartheta +\cos^{3}\vartheta \cdot \cos\phi \right) \end{array}$$

The quantity $\tilde{G}$ follows formally from $\tilde{E}$ by permutation of the variables (ϑ, *ϕ*):

$$\tilde{G}=a_{\phi }{\cdot a}_{\phi }=\frac{\cos^{2}\phi }{{\left(\cos^{2}\vartheta -\sin^{2}\phi \right)}^{3}}\left(\cos^{4}\vartheta +\sin^{2}\phi \cdot \sin^{2}\vartheta \right)$$
